# Supplementary material for: Dialdehyde Cellulose Solution as Reducing Agent: Preparation of Uniform Silver Nanoparticles and In Situ Synthesis of Antibacterial Composite Films with High Barrier Properties
Source: Molecules. 2023 Mar 26;28(7):2956. doi: 10.3390/molecules28072956 (PMC10095822; doi:10.3390/molecules28072956)
Supplement: Supplementary file 1 [file molecules-28-02956-s001.zip › molecules-2275331-supplementary.pdf]

Supplementary Material

Dialdehyde Cellulose Solution as Reducing Agent: Preparation of Uniform Silver Nanoparticles and In Situ Synthesis of Antibacterial Composite Films with High Barrier Properties

Jinsong Zeng [1,2], Xinyi Xiong [1,2], Fugang Hu [1,2], Jinpeng Li [1,2] and Pengfei Li [1,2,3,*]

[1] Plant Fibril Material Science Research Center, State Key Laboratory of Pulp and Paper Engineering, School of Light Industry and Engineering, South China University of Technology, Guangzhou 510640, China
[2] Guangdong Provincial Key Laboratory of Plant Resources Biorefinery, Guangzhou 510006, China
[3] School of Environment and Energy, South China University of Technology, Guangzhou 510640, China
[*] Correspondence: felpf@scut.edu.cn; Tel.: +86-18354219786

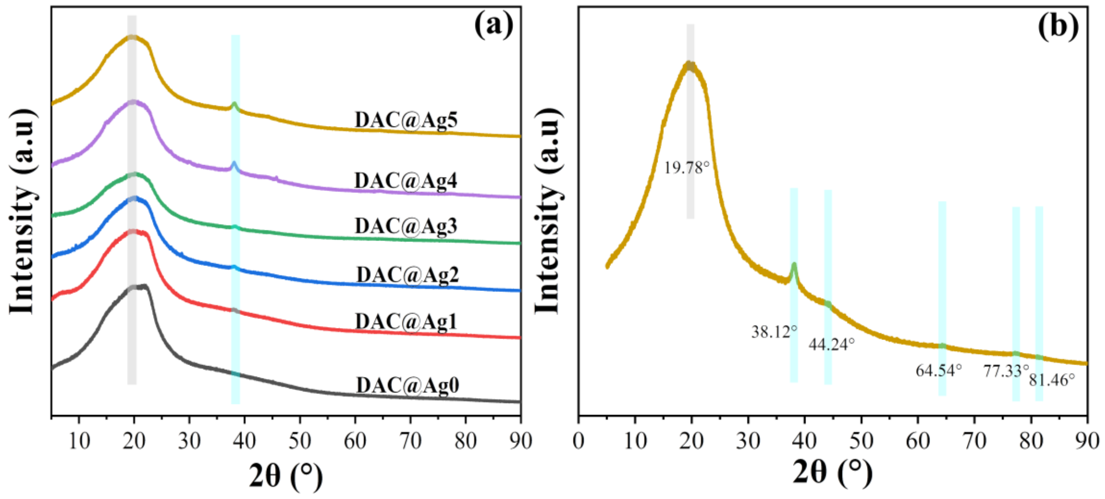

Figure S1. (a) XRD spectra of DAC@Ag0-DAC@Ag5 composite films; (b) enlarged view of the spectra of DAC@Ag5 composite film.

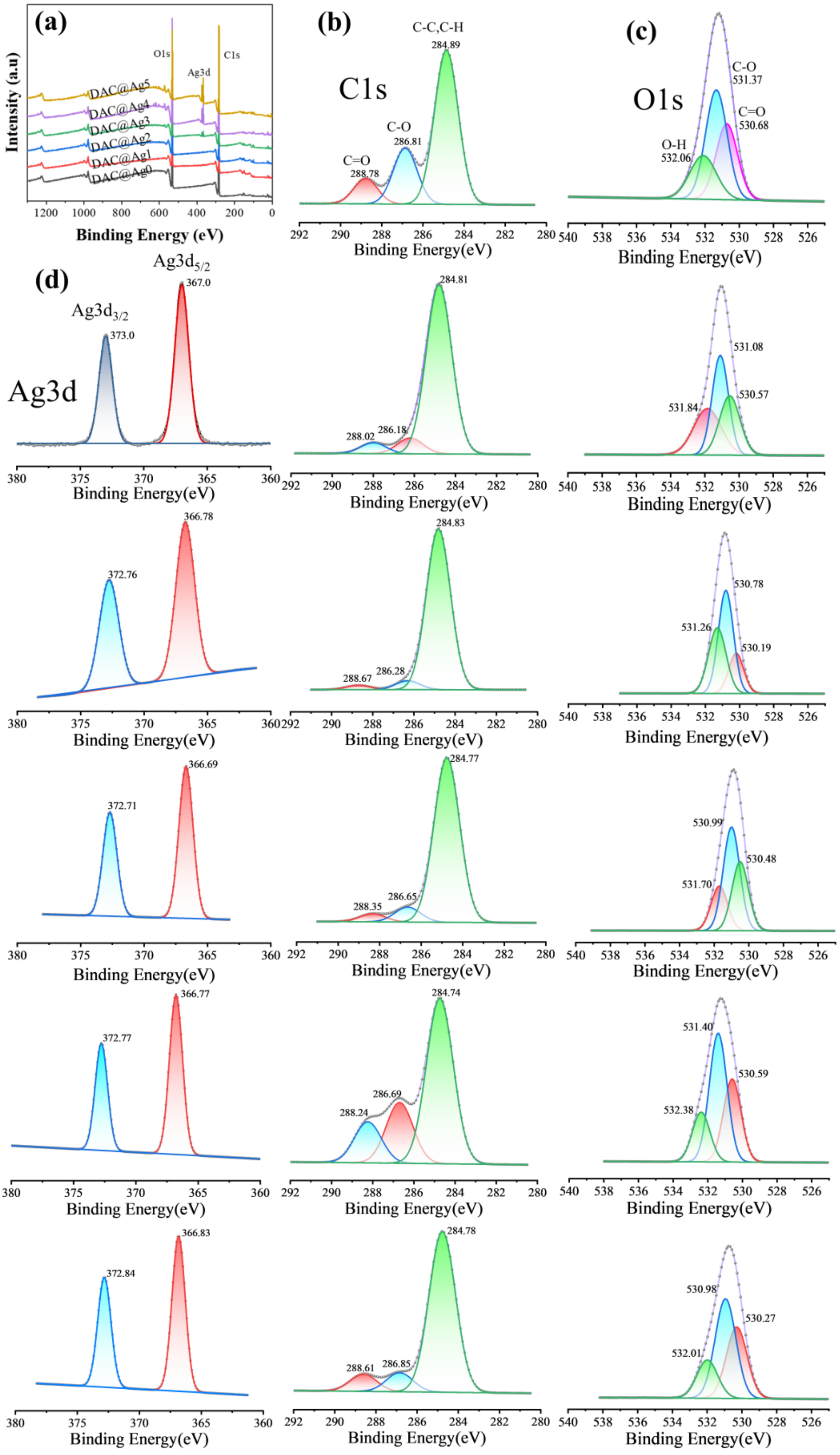

Figure S2. XPS spectra of DAC@Ag0-DAC@Ag5 composite films: (a) full scan spectrum, (b) C1s region, (c) O1s region and (d) Ag3d region.
